# 60 is the new 40: preparing for better bone health in later life

**DOI:** 10.3389/fragi.2025.1490124

**Published:** 2025-03-19

**Authors:** Leo Westbury, Kamran Gaba, Gregorio Bevilacqua, Nicholas Fuggle, Elaine Dennison

**Affiliations:** MRC Lifecourse Epidemiology Centre, University of Southampton, Southampton, United Kingdom

**Keywords:** bone, calcium, fracture, cardiovascular, cohort

## Abstract

**Objective:**

In this study we evaluated associations between nutritional factors, including calcium supplementation, and outcomes of fracture and cardiovascular mortality. We chose to report both outcomes as an illustration of the importance of nutritional factors in midlife to heart disease as this may be more impactful for supporting behavior change strategies, particularly in men.

**Methods:**

This study was nested in the Hertfordshire Cohort Study, a community dwelling cohort of 2,997 adults (47% women) who were extensively phenotyped at baseline and followed up for 20 years using Hospital Episode Statistics linkage.

**Results:**

Mean (SD) age at baseline was 65.7 (2.9) among men and 66.6 (2.7) among women. There was some evidence that better diet quality was related to reduced risk of hip fracture after adjustment for sex (hazard ratio (95% CI): 0.82 (0.67, 1.00) per SD higher prudent diet score). Dietary calcium intake was not associated with either any fracture or hip fracture. Taking calcium supplements was associated with an increased risk of any fracture, possibly because of reverse causality as calcium supplements will typically be prescribed following an osteoporotic fracture. A higher dietary calcium intake was protective against cardiovascular-related mortality, while taking calcium supplements led to no excess risk (p = 0.870). Higher prudent diet scores, indicative of better diet quality, were related to other beneficial lifestyle choices such as reduced odds of ever smoking [odds ratio (95% CI) per SD higher diet score: 0.69 (0.63,0.74)], and higher physical activity (SD difference in physical activity score per SD higher diet score: 0.06 (0.02,0.10)).

**Conclusion:**

We have demonstrated the commonality of lifestyle factors to adverse clinical outcomes of fracture and heart disease in older adults. These data might be used in behavior change strategies aimed to improve nutrition and linked factors in midlife.

## Introduction

Osteoporosis is a chronic condition characterized by decreased bone density and quality, leading to increased bone fragility and susceptibility to fractures (Cole et al., 2008). These fractures are a significant public health concern, particularly among older adults (Cole et al., 2008). The most common sites for osteoporotic fractures include the hip, spine and wrist, with potentially devastating consequences such as pain, disability, loss of independence and even mortality (Cole et al., 2008). The burden of osteoporotic fractures is substantial and expected to grow as the population ages (Clynes et al., 2020).

Adequate intake of calcium, vitamin D, protein, and other essential nutrients is important for supporting bone formation, remodeling and repair. Therefore, promoting healthy dietary habits is paramount for reducing the risk of osteoporosis (Liu et al., 2024). Calcium is well known as a critical nutrient for bone health, serving as a key building block for bone tissue. Despite its importance, many adults fail to meet recommended calcium intake levels, primarily due to dietary choices; studies indicate that a significant proportion of adults consume diets lacking in calcium-rich foods, such as dairy products, green vegetables and fortified foods (Liu et al., 2024). Other nutrients associated with bone health such as vitamin D, vitamin K, potassium, magnesium and phosphorus are typically derived from a good intake of fresh fruit and vegetables, nuts, lean proteins and fortified foods (Peng et al., 2024).

There is evidence that poor health behaviors cluster, with poor diet quality more common among individuals with other poor health behaviors such as current smoking, low levels of physical activity and high alcohol consumption, which are also risk factors for osteoporosis (Zhang et al., 2016). Retirement represents a pivotal life stage characterized by transitions in daily routines, social roles and health behaviors and hence offers an opportune time for individuals to reassess and improve their lifestyle habits, including diet and physical activity (Dalgas et al., 2024).

However, any behavior intervention can be challenging and demonstrating the importance of lifestyle to risk of subsequent disease is important in discussions, allowing people to make an accurate self-perception of risk and motivating individuals to make lifestyle change. Understanding risk of osteoporotic fracture is a particular challenge in men–despite a lifetime risk of 10%–25% and evidence of higher morbidity and mortality among men who sustain a hip fracture (Adler, 2014), awareness of bone health is often especially poor in men. For example, in a recent qualitative analysis of male participants aged 55–68 years from the Hertfordshire Intergenerational Study, some perceived osteoporosis as a disease that only affects women with many reporting much greater understanding of risk factors for cardiovascular disease than osteoporotic fracture (Bevilacqua et al., 2024; Hammond et al., 2024).

The aim of this study was therefore to evaluate relationships between lifestyle and fracture (any fracture and at the hip) among men and women followed for 20 years, contrasting fracture associations between lifestyle factors and mortality from cardiovascular disease. We hoped to use this analysis to inform educational material to be shared with health professionals working with people in their seventh decade. Specifically, we chose to assess lifestyle against the outcomes of both fracture and cardiovascular-related mortality as self-perception of risk is often more developed for heart disease among men and hence may be more persuasive in behavior change interventions.

## Materials and methods

The Hertfordshire Cohort Study (HCS) is a research study involving 2,997 individuals born in Hertfordshire, United Kingdom, between 1931 and 1939. Of these participants, 53% were men and 47% were women. These individuals were still residing in Hertfordshire during the period of 1998–2004. During this time, they participated in a home interview and underwent a health assessment at a clinic. The HCS was ethically approved by the Hertfordshire and Bedfordshire Local Research Ethics Committee. All participants provided written informed consent for the assessments conducted at the clinic and for future access to their medical records by researchers. Further details about the HCS have been previously published (Syddall et al., 2019).

During the home interview, information was collected from participants by a researcher-administered questionnaire. This included their smoking habits, any prior diagnoses of diabetes (excluding pregnancy-related cases) and hypertension. Physical activity was assessed from responses to questions about the frequency and duration of gardening, housework, climbing stairs and carrying loads in a typical week. A standardised activity score ranging from 0–100 was then calculated. The questionnaire had been designed specifically to characterise the level of physical activity in the elderly community dwelling population (Dallosso et al., 1988).

Diet was assessed using a food frequency questionnaire (FFQ) (Robinson et al., 2013). Foods were categorised into 51 groups based on their type and nutrient composition. Principal component analysis of the reported weekly frequencies of consumption of the food groups was used to describe the dietary patterns of participants. The first component described a “prudent” dietary pattern that follows recommendations for a healthy diet, characterised by high consumption of fruit, vegetables, whole-grain cereals and oily fish, with low consumption of white bread, chips, sugar and full-fat dairy products. A prudent diet score was calculated using the coefficient of each food group multiplied by the reported frequency of consumption of the food group, with the sum of these values providing a single score for each participant. Thus, a participant with a higher prudent diet score generally had a higher consumption of healthier items (fruit, vegetables, whole-grain cereals and oily fish) and a lower consumption of less healthy items (white bread, chips, sugar and full-fat dairy products) compared to a participant with a lower prudent diet score. The prudent diet score was used as an index of diet quality (Bloom et al., 2021). Dietary calcium intake was also assessed using this FFQ. We also recorded intake of supplements/medications containing calcium.

At the clinic visit, measurements of waist and hip circumferences were taken to an accuracy of 0.1 cm, which were then used to calculate waist-to-hip ratio. High waist-to-hip ratios were those greater than values recommended by the World Health Organization (≥0.90 among men and ≥0.85 among women) (World Health Organization, 2008). For those who hadn’t been previously diagnosed with diabetes, a 2-h fasting oral glucose tolerance test (OGTT) was conducted using 75 g of anhydrous glucose. Diabetes mellitus was then classified based on World Health Organisation guidelines (Alberti and Zimmet, 1998). Overall diabetes status was determined by cross-referencing self-reported diabetes status with medication data and the results from the OGTT. Resting blood pressure was determined by taking the average of three readings using a Dinamap Model 8,101 (GE Medical Systems, Slough, United Kingdom). Hypertension was defined as an average systolic pressure of 160 mmHg or higher and/or a diastolic pressure of 90 mmHg or higher, or if the participant was currently using prescribed anti-hypertensive medication.

### Ascertainment of subsequent adverse health outcomes

These events were identified using mortality and Hospital Episode Statistics (HES) data. The necessary permissions to access these data from HCS participants, spanning from the study baseline to 31 December 2018, were granted by NHS Digital and the Ethics and Confidentiality Committee of the National Information Governance Board. The process of linking the HCS cohort with HES data has been previously outlined (Rambukwella et al., 2024). The extracted HES data for each participant contained details about hospital admissions, including diagnoses coded to ICD-10. Adverse health events were defined according to the ICD-10 codes as outlined in Supplementary Table 1.

### Healthy conversation skills intervention study nested within the Hertfordshire Cohort Study

From November 2019 to March 2020, 176 participants were visited at home; 89 were randomised to the control group and received a healthy living leaflet, and 87 in the intervention group were interviewed using Healthy Conversation Skills at the visit, with follow-up telephone calls at 1, 3, 6 and 9 months. Healthy Conversation Skills is a communication approach used by health professionals that involves asking open-ended questions, active listening, and goal setting to empower individuals to make informed health decisions and positive behavioral changes (Hollis et al., 2021). Outcomes assessed by researcher-administered questionnaire at the visit, and at a 1-year follow-up through postal questionnaire included: physical activity, diet quality, appetite, SF-36 physical function, general self-efficacy, and social isolation. Longitudinal changes in outcomes from the visit to the 1-year follow-up were compared between trial arms. Detailed information on this intervention study has been published previously (Zhang et al., 2024).

### Statistical analysis

Characteristics of participants at the study baseline (1998–2004) and subsequent health events during the follow-up period were summarized using descriptive statistics. Time-to-first event Cox regression was used to examine exposures in relation to the adverse health events. Exposures included: age; smoking (ever vs. never); high alcohol intake (units per week: > 21 men, > 14 women); prudent diet score; dietary calcium intake; taking calcium supplements/medications (yes/no); physical activity; high waist-to-hip ratio; hypertension; and diabetes. Adverse health events included: any fracture; hip fracture; and cardiovascular-related mortality. Exposures that were statistically significant were then included in mutually-adjusted models. Stata (release 17.0) was utilized for statistical analysis with p-values of less than 0.05 being considered statistically significant. Similar associations were observed among men and women (data not shown) so sex-adjusted associations are presented among the pooled sample of men and women for all analyses.

Supplementary Analyses included examining prudent diet score in relation to ever smoking and high alcohol intake using sex-adjusted logistic regression, and in relation to physical activity using sex-adjusted linear regression.

## Results

### Participant characteristics

Table 1 outlines the participant characteristics of the analysis sample. Mean (SD) age at baseline was 65.7 (2.9) among men and 66.6 (2.7) among women. During follow-up, the proportion of participants who experienced each of the adverse health events were as follows: 9% of men and 22% of women were recorded as sustaining a fracture over follow up; 2% of men and 5% of women were recorded as having suffered a hip fracture. A record of cardiovascular-related mortality was made in 11% of men and 5% of women.

**TABLE 1:** Participant characteristics.

Participant characteristic [mean (SD), median (lower quartile, upper quartile), or %]	Men (n = 1,579)	Women (n = 1,418)
Characteristics at baseline (1998–2004)		
Age (years)	65.7 (2.9)	66.6 (2.7)
Ever smoked regularly	67%	39%
High alcohol intake (units per week: > 21 men, > 14 women)	22%	5%
Prudent diet score	−0.6 (2.1)	0.7 (1.7)
Dietary calcium intake (g/week)	8.5 (2.4)	7.8 (2.4)
Taking calcium supplement/medication	6%	16%
Dallosso physical activity score	60.9 (15.3)	59.0 (15.7)
Waist circumference (cm)	100.7 (10.5)	92.3 (12.6)
Hip circumference (cm)	104.1 (7.2)	108.0 (10.8)
Waist-to-hip ratio	0.97 (0.06)	0.85 (0.06)
High waist-to-hip ratio [≥0.90 (men), ≥0.85 (women)]	88%	53%
Hypertension	40%	41%
Diabetes	15%	14%
Mortality events during follow-up		
Death due to cardiovascular cause	11%	5%
Types of hospital admission during follow-up (ever had)		
Any fracture	9%	22%
Hip fracture	2%	5%

Follow-up period lasted from baseline (1998–2004) until 31st December 2018.

Associations between baseline participant characteristics and incident fracture (any) and hip fracture are presented in Table 2, and associations regarding cardiovascular-related mortality are presented in Table 3. In this cohort, there was some evidence that better diet quality was related to reduced risk of hip fracture after adjustment for sex (hazard ratio (95% CI): 0.82 (0.67,1.00) per SD higher prudent diet score). Dietary calcium intake was not associated with either any fracture or hip fracture. Taking calcium supplements was associated with an increased risk of any fracture, possibly because of reverse causality as calcium supplements will typically be prescribed following an osteoporotic fracture. In contrast, a higher dietary calcium was protective against cardiovascular-related mortality, while taking calcium supplements led to no excess risk (p = 0.870).

**TABLE 2:** Hazard ratios for any fracture and hip fracture according to each exposure.

Exposure	Any fracture				Hip fracture			
	Sex-adjusted		Mutually-adjusted		Sex-adjusted		Mutually-adjusted	
	Hazard ratio (95% CI)	P-value	Hazard ratio (95% CI)	P-value	Hazard ratio (95% CI)	P-value	Hazard ratio (95% CI)	P-value
Age (z-score)	**1.28 (1.17,1.41)**	**<0.001**	**1.26 (1.15,1.39)**	**<0.001**	**1.61 (1.33,1.96)**	**<0.001**	**1.57 (1.29,1.91)**	**<0.001**
Ever smoked regularly	**1.24 (1.03,1.50)**	**0.025**	**1.25 (1.03,1.51)**	**0.022**	1.29 (0.88,1.88)	0.193		
High alcohol intake	1.07 (0.79,1.46)	0.665			**1.76 (1.03,3.00)**	**0.039**	**1.85 (1.09,3.16)**	**0.024**
Prudent diet score (z-score)	0.92 (0.83,1.02)	0.100			0.82 (0.67,1.00)	0.052		
Dietary calcium intake (z-score)	0.98 (0.89,1.07)	0.639			0.99 (0.82,1.19)	0.887		
Taking calcium supplement/medication	**1.33 (1.03,1.73)**	**0.028**	**1.38 (1.06,1.78)**	**0.016**	1.01 (0.57,1.77)	0.982		
Physical activity (z-score)	**0.84 (0.77,0.93)**	**<0.001**	**0.87 (0.79,0.95)**	**0.003**	0.89 (0.74,1.08)	0.241		
High waist-to-hip ratio	1.20 (0.97,1.47)	0.089			1.12 (0.74,1.70)	0.578		
Hypertension	**1.27 (1.05,1.52)**	**0.012**	1.18 (0.98,1.42)	0.083	**1.81 (1.25,2.61)**	**0.002**	**1.64 (1.13,2.38)**	**0.009**
Diabetes	1.08 (0.83,1.41)	0.559			1.18 (0.70,1.98)	0.527		

Hazard ratios were estimated from Cox regression models; hazard ratios are presented per SD increase in each exposure or for the presence versus absence of each exposure. Mutually-adjusted models also included sex as an adjustment. Statistically significant associations (p < 0.05) are highlighted in bold. High alcohol intake: units per week > 21 men, > 14 women.

**TABLE 3:** Hazard ratios for cardiovascular-related mortality according to each exposure.

Exposure	Sex-adjusted		Mutually-adjusted	
	Hazard ratio (95% CI)	P-value	Hazard ratio (95% CI)	P-value
Age (z-score)	**1.45 (1.27,1.66)**	**<0.001**	**1.38 (1.21,1.58)**	**<0.001**
Ever smoked regularly	**1.39 (1.06,1.82)**	**0.018**	1.29 (0.98,1.70)	0.066
High alcohol intake	1.04 (0.74,1.46)	0.825		
Prudent diet score (z-score)	0.89 (0.79,1.02)	0.092		
Dietary calcium intake (z-score)	**0.81 (0.72,0.92)**	**0.002**	**0.85 (0.74,0.96)**	**0.012**
Taking calcium supplement/medication	1.04 (0.67,1.62)	0.870		
Physical activity (z-score)	**0.74 (0.65,0.83)**	**<0.001**	**0.79 (0.69,0.89)**	**<0.001**
High waist-to-hip ratio	1.30 (0.92,1.85)	0.132		
Hypertension	**2.30 (1.78,2.98)**	**<0.001**	**1.91 (1.46,2.50)**	**<0.001**
Diabetes	**1.92 (1.41,2.61)**	**<0.001**	**1.46 (1.07,2.01)**	**0.017**

Hazard ratios were estimated from Cox regression models; hazard ratios are presented per SD increase in each exposure or for the presence versus absence of each exposure. Mutually-adjusted models also included sex as an adjustment. Statistically significant associations (p < 0.05) are highlighted in bold High alcohol intake: units per week > 21 men, > 14 women.

**FIGURE 1:**
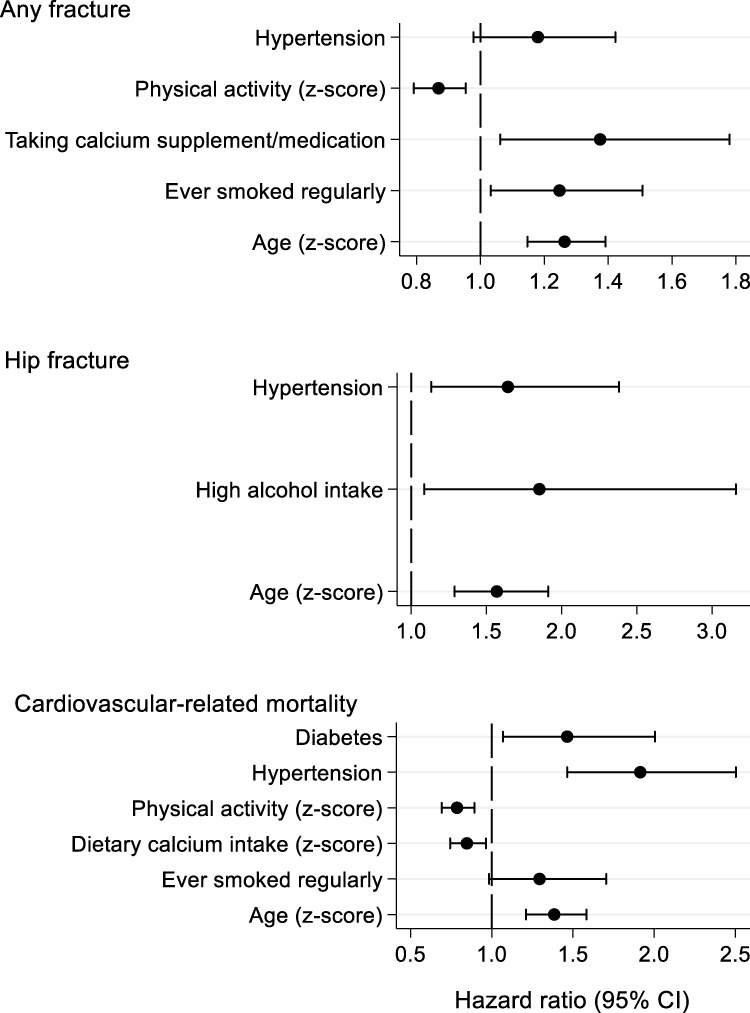
Mutually-adjusted associations between baseline participant characteristics and risk of incident adverse health events. Hazard ratios were estimated from Cox regression models; hazard ratios are presented per SD increase in each exposure or for the presence versus absence of each exposure.

Other lifestyle factors and comorbidities were associated with both risk of any fracture and cardiovascular-related mortality. For example, ever smoking [hazard ratio (95% CI): 1.25 (1.03, 1.51)] and lower physical activity (0.87 (0.79,0.95) per SD higher level) at baseline were related to increased risk of incident fracture (any) in a mutually-adjusted Cox model that also accounted for age, sex, hypertension and whether participants were taking calcium supplements/medications (Figure 1). Hypertension at baseline was related to increased risk of hip fracture [1.64 (1.13,2.38)] after adjustment for age, sex and alcohol intake (Figure 1). By comparison, ever smoking was related to increased risk of cardiovascular-related mortality in sex-adjusted analysis and lower physical activity and hypertension were related to increased risk of cardiovascular-related mortality in both sex-adjusted and mutually-adjusted analysis (Figure 1).

Associations between prudent diet score and smoking status, alcohol consumption and physical activity are presented in Supplementary Table 2. Higher prudent diet scores, indicative of better diet quality, were related to reduced odds of ever smoking [odds ratio (95% CI) per SD higher diet score: 0.69 (0.63,0.74)], and higher physical activity [SD difference in physical activity score per SD higher diet score: 0.06 (0.02,0.10)]. Associations between prudent diet score and high alcohol intake were weak (p = 0.542).

## Discussion

We have found that a more “prudent” diet was associated with a weak protective effect against hip fracture (p = 0.052) and mortality from cardiovascular disease (p = 0.092) over 20 years of follow-up. While dietary calcium intake was associated with a protective effect against cardiovascular-related mortality in this sample, calcium supplement use was associated with increased risk of fracture, likely from reverse causation. Higher (or “better”) diet quality was associated with other lifestyle factors positively associated with bone health, namely, higher physical activity and never smoking, whilst associations with alcohol consumption were weaker. There was a very similar pattern of benefit from these factors for cardiovascular outcomes in the same cohort. We hope demonstration of the association of good diet in midlife with both hip fracture and cardiovascular-related mortality will motivate individuals to make changes for the better. Even if it is hard to convince some groups of the benefits of change for bone health, illustration of rewards for cardiovascular disease may prove more persuasive.

There are of course several limitations and strengths to this work. We chose to undertake this analysis in this cohort as it is well phenotyped and follow up was available for over 20 years, through linkage to Hospital Episode Statistics, which provides data on all NHS hospital admissions. Information on admission to private hospitals were not available to us. Participants in the Hertfordshire Cohort Study have previously been shown to be representative of the general population with regard to smoking and anthropometric characteristics but the population is 100% Caucasian (Syddall et al., 2005) and all study participants were born in Hertfordshire and lived there throughout their adult life. Dietary habits were established through use of a food frequency questionnaire but serum measures of vitamins relevant to bone health were not available. Finally, we do not know the timeline of calcium supplementation to fracture, so are unable to confirm our suggested explanation of reverse causation for the association between calcium supplement use and fracture.

Potential risks to calcium supplementation for bone health have proved to be controversial over the years. After a report that suggested that it may be associated with excess cardiovascular risk in a *post hoc* analysis in a New Zealand trial setting (Bolland et al., 2008), many patients were reluctant to continue with their calcium supplementation. Subsequent analyses, including in the large UK Biobank cohort were reassuring (Harvey et al., 2018) but concerns remained among some, so these data provide further reassurance regarding the link between calcium supplementation and cardiovascular risk. This is valuable as although dietary calcium intake (rather than supplements) has never been linked with cardiovascular risk, not everyone can attain recommended levels of calcium intake (at least 700 mg daily) through diet alone.

The observed protective effect of a higher dietary calcium intake on risk of cardiovascular-related mortality was interesting. Several other large epidemiological studies have suggested a protective effect of higher calcium intake on heart disease, with some evidence that this effect may be greater in hypertensive patients through an effect on vascular resistance (Lee et al., 2024; Yazdanpanah et al., 2024). Other explanations include effects on lipid levels, glucose handling and inflammation. The benefit of a high calcium intake for both bone and cardiovascular health is an important message to share with individuals when discussing dietary change.

Other than calcium intake, there is an extensive literature around the associations of nutrition with bone health, with recent reviews summarizing the evidence for the associations of a diet adequate for vitamin D and vitamin K (Rusu et al., 2024), adequate protein intake (Kędzia et al., 2023) and avoidance of a high fat diet with low nutritional value (Dole et al., 2024). The diet quality measure we use in this study is a broad reflection of dietary patterns overall and our study supports evidence from other studies. Interestingly, recent work has highlighted the interaction between physical activity and nutrition in promoting better bone health in older adults (Dolan et al., 2024). This systematic review highlighted the heterogeneity of research in this area but reported a meta-analysis that indicated that calcium intake reduced exercise-induced increases in the bone turnover marker CTX-1. Narrative synthesis suggests that carbohydrate supplementation may support bone during acute exercise, via reducing exercise-induced increases in CTX-1. Conversely, a low-carbohydrate/high-fat diet appeared to produce the opposite effect. The researchers therefore suggested that low energy availability may amplify the CTX-1 response to exercise, but it is unclear whether this is directly attributable to energy availability or to the lack of specific nutrients, such as carbohydrate. These data are important as they highlight the need to consider physical activity and nutrition together when developing behavioural strategies to promote better bone health in older adults.

Comorbidities such as hypertension (including use of antihypertensives) were associated with both fracture and mortality from cardiovascular disease in our cohort. The association between hypertension and heart disease is well established (Ebrahim and Smith, 1997), but the literature between blood pressure and bone health is less extensive (Canoy et al., 2022). A high proportion of our study participants were taking medication and we speculate that the association may in part be explained by postural hypotension increasing the propensity for falls. A systematic review and meta-analysis highlighted a positive association between thiazide diuretics and bone health, suggesting that this effect may not be uniform across all medication classes (Desbiens et al., 2022).

Behaviour change is widely acknowledged to be challenging. Healthy Conversation Skills is one way for healthcare providers to opportunistically support individuals by asking open-ended (“open discovery”) questions, reflecting on practice, listening more than giving information and supporting SMARTER (Specific, Measurable, Action-oriented, Realistic, Timed, Evaluated, Reviewed) goal setting (Hollis et al., 2021). While Healthy Conversation Skills has been shown to be applicable, feasible and acceptable in a wide range of situations (Hollis et al., 2021), it is less commonly used in older adults. We recently undertook a modest study in a subgroup of the Hertfordshire Cohort study population of mean age 83 years, demonstrating that it is viable to utilise Healthy Conversation Skills via telephone to promote healthier lifestyles in older adults (Zhang et al., 2024). In our study of 176 participants randomised to the intervention group (received a Healthy Conversation Skills intervention) or the control group (received an information leaflet), we found that among women, there was a tendency for greater increases in diet quality in the intervention group compared to the control group, while among men, there was a tendency for reduced decline in self-reported physical function in the intervention group compared to the control group. Larger appropriately powered studies to determine the efficacy of such an intervention are now required, with longer follow up periods to evaluate the sustainability of such an approach.

## Conclusion

We have demonstrated associations between prudent diet and physical activity with outcomes of fracture and cardiovascular mortality over 20 years of follow-up. These associations can be used to highlight the benefits of behavior change to people in their seventh decade to improve both their cardiovascular and bone health.

## Data Availability

The data analyzed in this study is subject to the following licenses/restrictions: Data relating to this study cannot be shared due to consent restrictions.
