# Supplementary material for: 60 is the new 40: preparing for better bone health in later life
Source: Front Aging. 2025 Mar 19;6:1490124. doi: 10.3389/fragi.2025.1490124 (PMC11962032; doi:10.3389/fragi.2025.1490124)
Supplement: Supplementary file 1 [file Supplementaryfile1.pdf]

Supplementary Table 1: ICD-10 codes used to define adverse health events

Adverse health event	ICD-10 codes
**Cardiovascular-related mortality**	I10-I79
**Any fracture**	M80: Osteoporosis with pathological fracture<br>M84: Disorders of continuity of bone<br>S22: Fracture of rib(s), sternum and thoracic spine<br>S32: Fracture of lumbar spine and pelvis<br>S42: Fracture of shoulder and upper arm<br>S52: Fracture of forearm<br>S62: Fracture at wrist and hand level<br>S72: Fracture of femur<br>S82: Fracture of lower leg, including ankle<br>S92: Fracture of foot, except ankle<br>T02: Fractures involving multiple body regions<br>T08: Fracture of spine, level unspecified<br>T10: Fracture of upper limb, level unspecified<br>T12: Fracture of lower limb, level unspecified<br>M81: Osteoporosis without pathological fracture<br>M82: Osteoporosis in diseases classified elsewhere<br>M83: Adult osteomalacia<br>M90.7: Fracture of bone in neoplastic disease<br>S02: Fracture of skull and facial bones<br>S12: Fracture of neck<br>T90.2: Sequelae of fracture of skull and facial bones<br>T91.1: Sequelae of fracture of spine<br>T91.2: Sequelae of other fracture of thorax and pelvis<br>T92.1: Sequelae of fracture of arm
**Hip fracture**	S72.0 Fracture of neck of femur; Fracture of hip NOS<br>S72.1 Pertrochanteric fracture; intertrochanteric fracture; trochanteric fracture<br>S72.2 Subtrochanteric fracture

Supplementary Table 2: Associations between prudent diet score and other health behaviours (pooled and adjusted for sex)

Health behaviour	Odds ratio (95% CI)	P-value
Ever smoking (yes vs no)	0.69 (0.63,0.74)	<0.001
High alcohol intake (yes vs no)	1.03 (0.93,1.15)	0.542
Dallosso physical activity score (z-score)	0.06 (0.02,0.10)	0.002

High alcohol intake (units per week: > 21 men, > 14 women)

Odds ratio (95% CI) for health behaviour per SD increase in prudent diet score; for physical activity, estimates for difference in physical activity score per SD increase in diet score are shown
